# FGSE02, a Novel Secreted Protein in *Fusarium graminearum* FG-12, Leads to Cell Death in Plant Tissues and Modulates Fungal Virulence

**DOI:** 10.3390/jof11050397

**Published:** 2025-05-21

**Authors:** Zhigang Hao, Lei Pan, Jiaqing Xu, Chengxuan Yu, Jianqiang Li, Laixin Luo

**Affiliations:** 1 Department of Plant Pathology, China Agricultural University, Beijing 100193, China; 17810266056@163.com (Z.H.); jiaqingxu2020@163.com (J.X.); yu18846183781@163.com (C.Y.); lijq231@cau.edu.cn (J.L.); 2 Key Laboratory of Integrated Pest Management on Crops in Northwestern Oasis, Ministry of Agriculture and Rural Affairs, National Plant Protection Scientific Observation and Experiment Station of Korla, Xinjiang Key Laboratory of Agricultural Biosafety, Institute of Plant Protection, Xinjiang Uygur Autonomous Region Academy of Agricultural Sciences, Xinjiang, Urumqi 830091, China; 3 Sanya Institute of China Agricultural University, Sanya 572025, China; 4 Sanya Research Institution, Chinese Academy of Tropical Agriculture Sciences/Hainan Key Laboratory for Biosafety Monitoring and Molecular Breeding in Off-Season Reproduction Regions, Sanya 571101, China; 5 Center for Biosafety, Chinese Academy of Inspection and Quarantine, Sanya 572024, China; pp1181494002@163.com

**Keywords:** transcriptome, *Fusarium graminearum*, virulence-associated genes, effector protein

## Abstract

Fungal phytopathogens employ effector proteins and secondary metabolites to subvert host immunity. Effector proteins have attracted widespread interest in infection, especially for unknown, unreported genes. However, the type of protein remains much less explored. Here, we combined transcriptome analysis and functional validation to identify virulence-associated genes in *Fusarium graminearum* during fungi infection. A unique secreted protein, FGSE02, was significantly upregulated in the early infection stage. Proteomic characterization revealed that the protein contains a functional signal peptide but lacks known domains. The transient expression of FGSE02 in *Nicotiana benthamiana* induced rapid cell death, while gene knockout stains reduced fungal virulence without affecting growth. Our findings highlight FGSE02 as a key virulence factor, offering potential targets for disease control. Taken together, the results of this study identify a pathogenic factor and provide new insights into the development of green pesticides.

## 1. Introduction

The genus Fusarium represents a group of filamentous fungi with profound agricultural and economic impacts, collectively responsible for yield losses in staple grains and garden crops, such as wheat, maize, papaya, and tea [1,2,3,4,5]. Among these pathogens, *Fusarium graminearum* stands out as a major causal agent of Fusarium head blight (FHB) and ear rot [6,7,8], diseases that not only devastate grain production but also contaminate harvests with trichothecene mycotoxins [6,9], particularly deoxynivalenol (DON). DON accumulation in cereals poses severe health risks to humans and livestock, including immunosuppression and gastrointestinal disorders [10,11], while its phytotoxic effects accelerate host cell necrosis, facilitating fungal colonization [12]. Underscoring the urgency of understanding its pathogenic mechanisms, *F. graminearum* causes significant economic losses worldwide each year [13]. Therefore, elucidating the pathogenic mechanism is of paramount importance.

During infection, *F. graminearum* deploys a multifaceted array of virulence factors, including carbohydrate-active enzymes (CAZymes) that degrade plant cell walls, effector proteins that suppress host immunity, and secondary metabolites that disrupt cellular homeostasis [14,15]. Fungal pathogenicity in *F. graminearum* relies on a suite of secreted virulence factors that disrupt plant immunity through distinct molecular strategies [16]. Host-modulating proteins target critical defense components: FGL1 lipase undermines plant immunity by inhibiting synthase activity [16], whereas Arb93B arabinase (FGSG_03598) suppresses BAX-dependent plant cell death [17]. Nuclear-targeting FgNls1 disrupts chromatin through histone interaction [18], while chloroplast-localized Fg03600 alters photosynthetic electron transport via competitive binding with TaPGRL1/TaFd complexes [19]. Protease virulence factors demonstrate multifunctional suppression mechanisms; FgTPP1 endopeptidase attenuates chitin-induced MAPK signaling [20] and cell death, whereas FgEC1 promotes TaRBOHD degradation [21]. This pathogen synergistically deploys mycotoxins with proteinaceous effectors, exemplified by DON’s ribotoxic stress synergizing with virulence factors like Osp24 ubiquitination activator [22] and Fg12 ribonuclease [23]. Recent studies further identify necrosis-related effectors including Fg62-induced cell death elicitor [24] and subtilisin protease mediating host tissue maceration, though their molecular targets require elucidation [25]. Despite these advances, the functional characterization of *F. graminearum* effectors remains incomplete, highlighting critical knowledge gaps in fungal–plant interplay [26]. Although the genes involved in pathogenicity have been studied, there are few studies of pathogenicity-related genes in the *F. graminearum* genome of more than 12,000 genes [27], which is insufficient to fully analyze its pathogenic mechanism.

Recent advances in transcriptomics and bioinformatics have reshaped the identification of fungal virulence factors. RNA sequencing (RNA-seq) enables the genome-wide profiling of pathogen gene expression during infection, revealing stage-specific expression of effector and metabolic pathways [28,29,30]. For example, comparative transcriptomics of *Fusarium oxysporum* f. sp. cubense (Foc) strains demonstrated that broad-host-range isolates upregulate CAZymes and effector-like genes during colonization [31]. Similarly, in *Botrytis cinerea*, comprehensive analysis of RNA-seq and knockout mutants identified an unannotated gene essential for conidial germination and host penetration [32]. These studies highlight the potential of multi-omics approaches to uncover novel virulence determinants.

In this study, we combined RNA-seq analysis of *F. graminearum* during maize infection with systematic functional validation to identify FGSE02, a previously uncharacterized secreted protein. Transcriptomic profiling revealed FGSE02 as one of the most highly induced effector candidates during early infection. Bioinformatics analysis predicted a functional signal peptide but no conserved domains, suggesting its unique mode of infection. The transient expression of FGSE02 in *N. benthamiana* induced rapid cell death, while gene knockout weakens fungal virulence without compromising hyphal growth or sporulation. Our findings not only expand the known effector spectrum of *F. graminearum* but also provide a target for developing dsRNA or small-molecule bases in integrated pest management (IPM) systems.

## 2. Materials and Methods

### 2.1. Fungal Strains, Plant Materials, and Growth Conditions

The *F. graminearum* wild-type strain was isolated from infected maize in the Hexi Corridor, Gansu Province, China. Maize (*Zea mays* cv. B73) seeds were surface-sterilized with 1% sodium hypochlorite (2 min), rinsed three times with sterile deionized water, soaked for 4 h, and germinated on moist filter paper in the dark at 25 °C. *Escherichia coli* DH5α and *Agrobacterium tumefaciens* GV3101 were cultured in LB medium with appropriate antibiotics [27]. Fungal strains were maintained on potato dextrose agar (PDA) at 25 °C in the dark.

### 2.2. Infection Assays and Sample Collection

Germinated maize roots (2 cm length) were inoculated by immersion in *F. graminearum* spore suspensions (1 × 10^5^ spores/mL). Infected root tissues were harvested at 24, 48, and 72 h post-inoculation (hpi), flash-frozen in liquid nitrogen, and stored at −80 °C. For fungal mycelia samples, FG-12 was grown in potato dextrose broth (PDB) at 25 °C with shaking (160 rpm) for 3 days.

### 2.3. RNA Extraction, Library Construction, and Sequencing

Total RNA was extracted from infected roots and fungal mycelia using TRIzol reagent (Invitrogen, Waltham, MA, USA). RNA quality was assessed via agarose gel electrophoresis and NanoDrop spectrophotometry. Libraries were constructed using the TruSeq RNA Sample Prep Kit (Illumina, San Diego, CA, USA) and sequenced on the BGIseq500 platform (BGI, Shenzhen, China) with 150 bp paired-end reads (NCBI accession: PRJNA799676).

### 2.4. Transcriptome Data Processing and Analysis

Raw reads were filtered using fastp (v0.23.2) [33] and SOAPnuke (v2.0) [34] to remove adapters and low-quality sequences (Q20 > 95%). Clean reads were aligned to the *F. graminearum* genome using HISAT2 (v2.2.1) [35]. Gene expression levels were quantified with featureCounts (v2.0.3) [36], and differentially expressed genes (DEGs) were identified using DESeq2 (|log_2_FC| ≥ 1, *p* ≤ 0.05) [37]. Functional enrichment analysis (GO and KEGG) was performed using clusterProfiler (v4.0) [38].

### 2.5. FGSE02 Bioinformatics Characterization

FGSE02 homologs were identified via BLASTp (https://blast.ncbi.nlm.nih.gov/Blast.cgi?PROGRAM=blastp&PAGE_TYPE=BlastSearch&LINK_LOC=blasthome, accessed on 2 March 2021) with a threshold of >60% identity [39]. Conserved motif analysis of FGSE02 homologous proteins was predicted using MEME Suite (v5.5.0) [40]. Signal peptides, transmembrane domains, and GPI anchors were analyzed with SignalP-6.0 [41], TMHMM-2.0 [42], and PredGPI [43], respectively. Phylogenetic trees were constructed using MEGA-X (neighbor-joining method, 1000 bootstrap replicates) [44].

### 2.6. Yeast Secretion Assay

The FGSE02 signal peptide (first 120 bp of CDS) was cloned into the pSUC2 vector and transformed into *Saccharomyces cerevisiae* strain YTK12. Transformants were selected on CMD-W medium and validated for invertase secretion on YPRAA plates. Controls included YTK12 strains expressing Avr1b (positive), empty pSUC2 (negative) and Mg87 (negative).

### 2.7. FGSE02 Knockout and Complementation

The FGSE02 coding sequence was replaced with a hygromycin resistance cassette (HPH-HSV-tk) via homologous recombination. Flanking regions (1 kb upstream/downstream) were amplified and cloned into pBluescript SK(-). Protoplasts transformed with linearized constructs using PEG-mediated transformation. PCR and sequencing verified knockout mutants (ΔFGSE02) and complemented strains (ΔFGSE02-C).

### 2.8. Transient Expression in Nicotiana benthamiana

Full-length FGSE02 (with/without signal peptide) was cloned into the pBin-GFP2 vector and transformed into *Agrobacterium* GV3101. Bacterial suspensions (OD_600_ = 0.5) were infiltrated into leaves of 3-week-old *N. benthamiana*. Cell death was detected for 72 h. Controls included empty pBin-GFP2 constructs. Hydrogen peroxide (H₂O₂) detection was performed through 3,3′-diaminobenzidine (DAB) histochemical staining following established protocols with modifications [27].

### 2.9. Pathogenicity and Phenotypic Assays

The wild-type sample, ΔFGSE02, and ΔFGSE02-C were cultured on PDA for mycelial growth assessment (4 days, 25 °C). Sporulation was quantified in carboxymethyl cellulose (CMC) medium after 5 days. For pathogenicity assays, maize roots were inoculated with spore suspensions (1 × 10^5^ spores/mL), and disease severity (infected root length ratio) was measured at 72 hpi.

### 2.10. Statistical Analysis

Data were analyzed using GraphPad Prism 9. Significance was determined via ANOVA (*p* < 0.05). Error bars represent standard deviation (SD) of three biological replicates.

## 3. Results

### 3.1. Dynamics of F. graminearum Gene Expression During Maize Infection

The transcriptome sequencing of *F. graminearum* during early infection stages (24–72 h post-inoculation, hpi) revealed dynamic gene expression patterns. Sequencing quality was reliable, with Q20 scores > 95% and high mapping rates to the FG-12 genome (Table 1). Fungal reads in mixed (host–pathogen) samples increased progressively from 5.77% at 24 hpi to 42.29% at 72 hpi (Table 1), reflecting fungal colonization over time. Sample correlation analysis of transcriptomes confirmed strong intra-group reproducibility according to gene expression (Table S5, Figure S1).

### 3.2. Functional Enrichment of Differentially Expressed Genes (DEGs)

Differential expression analysis identified 4,232 genes (|log2FC| ≥ 1, *p* ≤ 0.05), with 1898 upregulated and 2334 downregulated during infection [27]. To explore the possible role of differently expressed genes (DEGs), DEGs were enriched by GO function and KEGG pathway enrichment, respectively.

**Figure 1:**
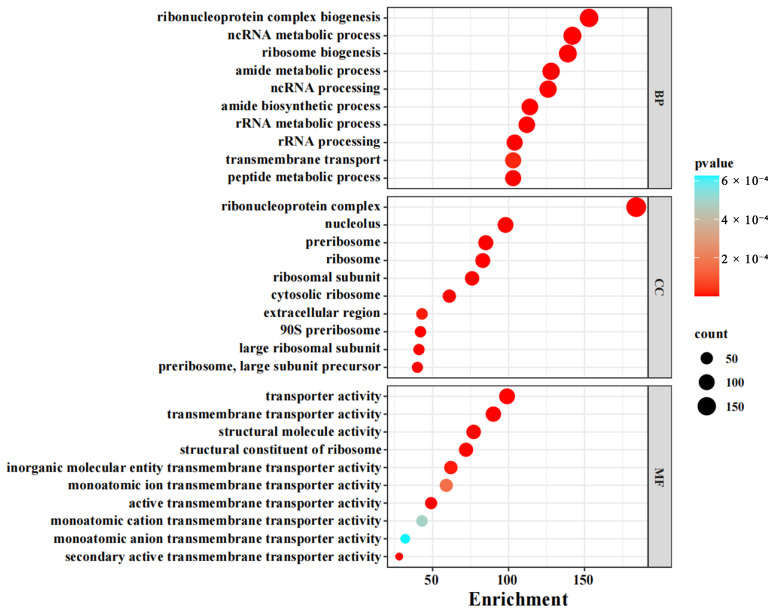
GO function enrichment of DEGs. Gene Ontology (GO), Molecular Function (MF), Cellular Component (CC), and Biological Process (BP). The size of the solid circle represents the number of genes, and the color represents the size of the confidence value (from blue to red, indicating that the confidence value is increasingly large).

GO function enrichment indicated that most of the DEGs are located in the ribosomal protein complexes, participate in ribosomal protein synthesis, and are involved in transporter activities. Gene Ontology (GO) enrichment highlighted ribosomal biosynthesis and transmembrane transport as dominant processes among DEGs (Figure 1).

**Figure 2:**
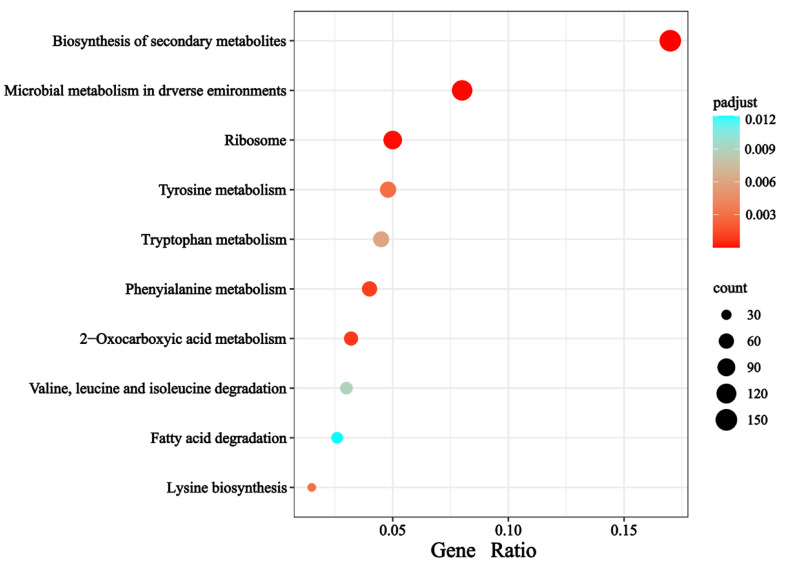
KEGG pathway enrichment of DEGs. Kyoto Encyclopedia of Genes and Genomes (KEGG). The size of the solid circle represents the number of genes, and the color represents the size of the confidence value (from blue to red, indicating that the confidence value is increasing).

Kyoto Encyclopedia of Genes and Genomes (KEGG) analysis showed that most of the DEGs are enriched in the synthesis of secondary metabolites, which indicates that secondary metabolites may play an important role in early infection (Figure 2).

In the early stage of *F. graminearum* infection, certain genes are significantly upregulated genes (>100-fold) and may play important roles (Table S1), such as two Common in Fungal Extracellular Membrane (CFEM) protein domains (FGMG_005117, FGMG_002274), a secreted protein (FGMG_000021), five members of the cytochrome P450 family (FGMG_001842, FGMG_000057, FGMG_005176, FGMG_011893, FGMG_005182), and five members of the ABC transport family (FGMG_005729, FGMG_004059, FGMG_012202, FGMG_012203, FGMG_004060) and so on.

### 3.3. Gene Analysis Related to Secondary Metabolite Biosynthesis

Fungal secondary metabolites serve as crucial virulence factors during host infection. Transcriptomic profiling revealed the significant upregulation of secondary metabolism-associated genes during early infection, including cytochrome P450 monooxygenases, ABC transporters, and specialized metabolite translocators, suggesting their critical role in fungal development. Notably, deoxynivalenol (DON)—which inhibits eukaryotic ribosomes and disrupts host membrane integrity through mitochondrial targeting—emerged as a key virulence factor. To systematically characterize DON biosynthesis, we employed antiSMASH-based genome mining coupled with expression validation, focusing on time-dependent activation patterns of the TRI gene cluster during fungi invasion.

**Figure 3:**
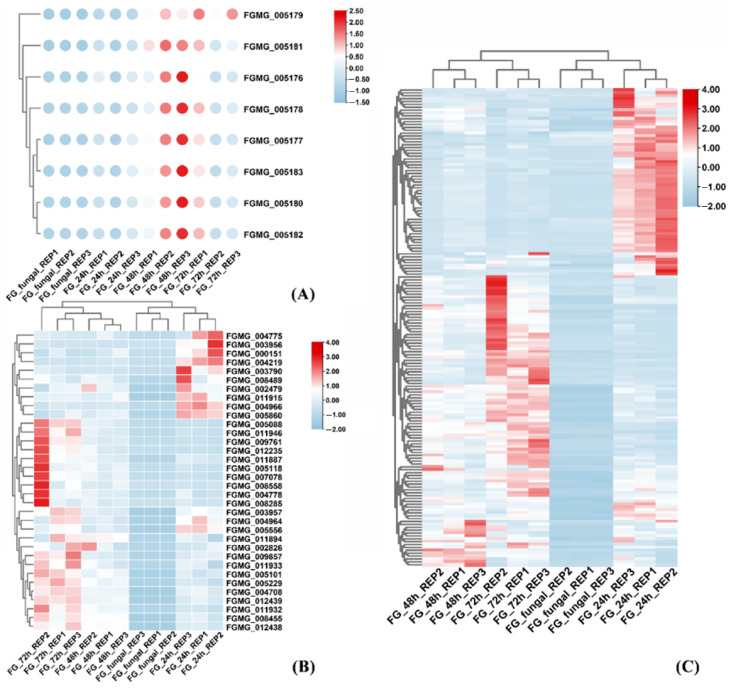
(**A**) The expression of DON toxin synthesis-related genes. (**B**) The expression of genes encoding carbohydrase. (**C**) The expression of genes associated with effector proteins.

Transcriptomic profiling revealed phase-specific induction patterns during *F*. *graminearum* infection, with four key DON biosynthesis-related genes (FGMG_005177 [isotrichodermin C-15 hydroxylase], FGMG_005178 [hypothetical protein], FGMG_005180 [trichodiene synthase], and FGMG_005182 [trichodiene oxygenase]) showing sharp upregulation during infection, suggesting their critical role in triggering toxin biosynthesis (Figure 3A). Notably, 162 genes were differentially upregulated at 24 h post-infection, including 12 glycosyl hydrolase (GH) family members (e.g., FGMG_008489, FGMG_011933, and FGMG_005088 exhibiting >100-fold induction) that potentially facilitate host cell wall degradation. Particularly striking was the >2000-fold induction of FGMG_007253, a glycosyltransferase (GT) family member, highlighting the coordinated action of carbohydrate-active enzymes in nutrient acquisition and infection progression (Figure 3B). Concurrently, 167/437 candidate effector proteins were upregulated during early pathogenesis (Figure 3C), including the unique effector FGSE02 (FGMG_000652), which lacks known domains but features a secretory signal peptide and shows infection-triggered expression (Table S1). These synergistic transcriptional changes elucidate a three-phase pathogenic strategy of *F. graminearum*: mycotoxin production, cell wall deconstruction, and effector-mediated host immune suppression.

### 3.4. Identification and Characterization of FGSE02

**Figure 4:**
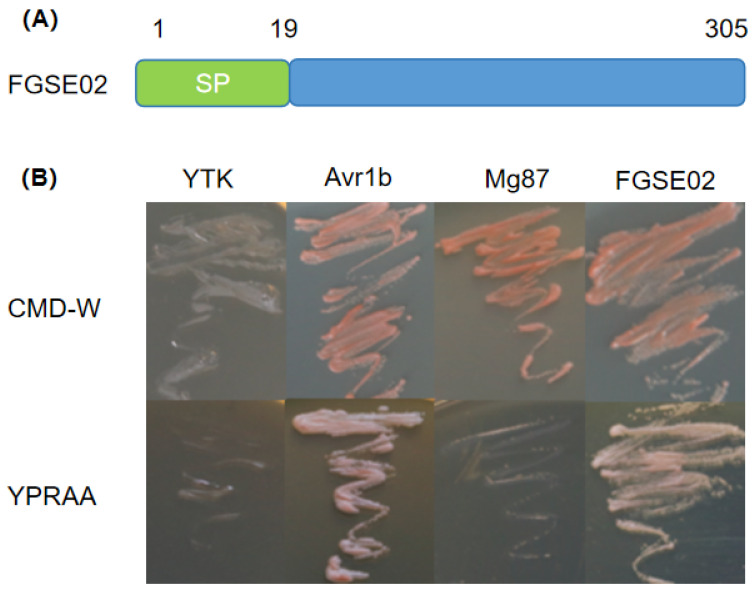
(**A**) The FGSE02 gene structure. SP: signal peptide. (**B**) Secretory function validation of signal peptide of FGSE02. YTK12: Yeast strain; Avr1b: positive control; Mg87: negative control.

Among 167 upregulated candidate effectors, FGSE02 (FGMG_000652) exhibited a >200-fold expression increase at 24 hpi (Table S1). FGSE02 is predicted to encode a 305-amino-acid secreted protein with a functional 19-residue N-terminal signal peptide but no transmembrane domains or GPI anchors (Figure 4A). Phylogenetic analysis showed that FGSE02 homologs (>60% identity) are unique to Fusarium species (Figure S1). Yeast secretion assays confirmed that the signal peptide’s activity, enabling YTK12 growth on YPRAA medium (Figure 4B).

As shown in Figure 4, FGSE02 could grow on YRPAA medium, as demonstrated by the positive control Avr1b. This experiment showed that the signal peptide sequence of FGSE02 has exocrine activity.

### 3.5. FGSE02 Induces Plant Cell Death and Modulates Pathogenicity

To delineate the molecular interplay between maize and *F. graminearum*, we engineered two expression constructs: one containing the full-length coding sequence (CDS) and another lacking the signal peptide (-SP) of the target gene, both fused to GFP in the pBin-GFP2 binary vector. These constructs were subsequently introduced into the *A. tumefaciens* strain GV3101 for transient expression assays in *N. benthamiana* leaves. This enabled the subcellular localization tracking and functional characterization of the candidate protein during host–pathogen interactions.

**Figure 5:**
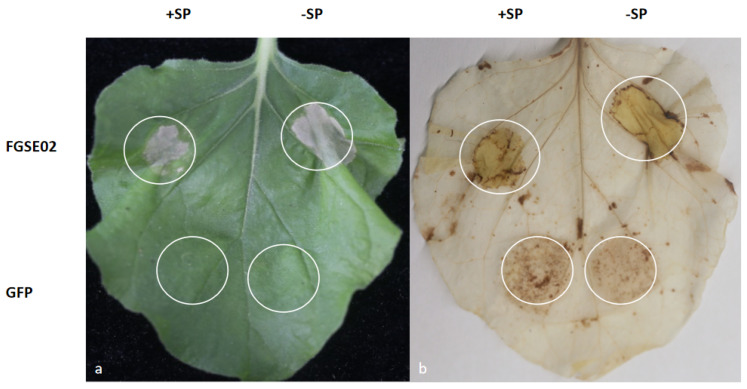
Transient expression of FGSE02 in *N. benthamiana* leaves by agro-infiltration (**a**). The presence of hydrogen peroxide via DAB staining (**b**). SP: signal peptide; GFP: green fluorescent protein. White circles (white dotted lines): infiltration zones in leaf tissues.

The transient expression of FGSE02 in *N. benthamiana* leaves led to cell death within 72 h, independent of its signal peptide (Figure 5). Histochemical analysis using DAB demonstrated pronounced H_2_O_2_ accumulation in line with FGSE02 expression, suggesting that this effector protein triggers a robust hypersensitive response (HR) characterized by oxidative burst in plant tissues.

We conducted a time-resolved analysis assay using an *F. graminearum* strain to illustrate the temporal transcriptional dynamics of FGSE02 during *F. graminearum*–host interaction.

**Figure 6:**
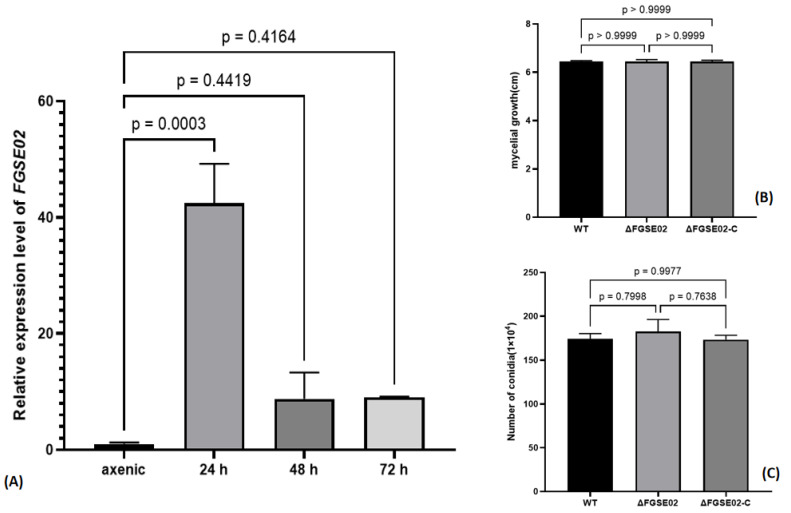
(**A**) Relative expression level of FGSE02 in fungal stage and at 24 hpi, 48 hpi, 72 hpi. (**B**) Vegetative growth rate of *F. graminearum* wild type strain FG-12, ΔFGSE02, and ΔFGSE02-C on PDA for 4 days. (**C**) Spore production statistics of *F. graminearum* wild type strain FG-12, ΔFGSE02, and ΔFGSE02-C in CMC medium for 5 days at 25 °C.

Quantitative RT-PCR analysis revealed interval-based expression of *FGSE02*, showing a sharp induction peak at 24 hpi (*p* < 0.001; one-way ANOVA) followed by stepwise reduction (Figure 6A). However, phenome analysis of ΔFGSE02 knockout strains demonstrated conserved adaptability, with radial growth rates on potato dextrose agar and conidiation capacity in carboxymethyl cellulose (CMC) liquid culture indistinguishable from wild-type counterparts (Figure 6A). This dichotomy suggests FGSE02 may function primarily during infection rather than fundamental fungal development.

To delineate the functional role of FGSE02 in fungal development and pathogen virulence competence, we generated targeted knockout mutants (ΔFGSE02) via PEG-mediated homologous recombination, coupled with genetic complementation strains (ΔFGSE02-C), enabling comparative phenotypic analysis of hyphal growth dynamics, sporulation efficiency, and plant infection capacity under controlled conditions.

**Figure 7:**
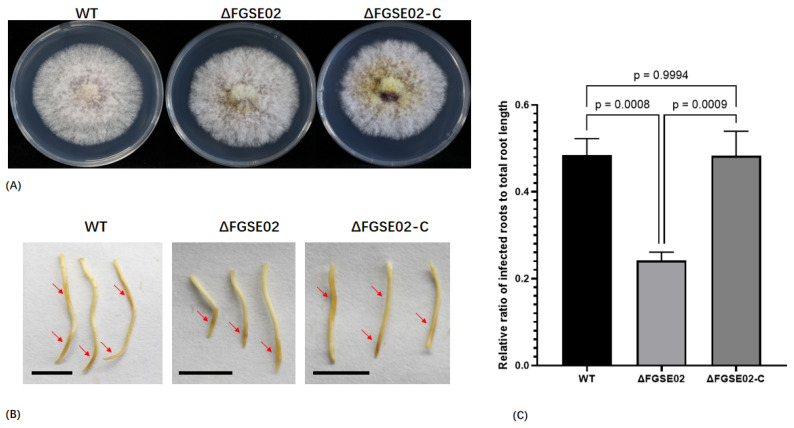
(**A**) Vegetative growth of *F. graminearum* wild type strain FG-12, ΔFGSE02, and ΔFGSE02-C on PDA for 4 days at 25 °C. (**B**) Pathogenic phenotypes caused by *F. graminearum* wild type strain FG-12, ΔFGSE02, and ΔFGSE02-C. Arrows indicate infection region; bar = 1 cm. (**C**) Relative ratio of length of infected root to total root.

Phenome analysis revealed conserved morphological features among the wild-type (WT), knockout mutant (ΔFGSE02), and complemented (ΔFGSE02-C) strains with the same hyphal growth rates on PDA media (Figure 6B) and equivalent sporulation capacities in liquid culture (Figure 6C). Notably, maize root infection assays demonstrated a reduction in necrotic lesion area in ΔFGSE02-infected plants compared to that in controls (Figure 7A–C), definitively identifying FGSE02 as an infection-specific effector pivotal for *F. graminearum* colonization in host tissue.

## 4. Discussion

The discovery of FGSE02 as an unreported secreted protein influencing *F. graminearum* pathogenicity provides critical insights into the molecular mechanisms underlying plant–fungal interactions. Our transcriptome analysis revealed that FGSE02, a single-exon gene encoding a signal peptide-containing protein, was upregulated during early infection. Its transient expression in *N. benthamiana* induced rapid cell death, while gene knockout reduced fungal virulence without affecting hyphal growth or sporulation. These findings position FGSE02 as a key virulence factor, distinct from growth-dependent effectors, and highlight its potential role in subverting plant immunity.

Notably, FGSE02 shares functional analogs with characterized fungal effectors such as Osp24 [22], fg12 [23], and PdCDIE1 [45], which suppress host defenses or induce necrosis to facilitate infection. However, unlike these proteins, FGSE02 lacks conserved domains or homology to known effector families, suggesting a unique mechanism of fungi invasion. This novelty aligns with emerging reports of effector proteins in phytopathogens, such as cassiicolin in *Corynespora cassiicola*, which similarly lack annotated domains but contribute to virulence [46]. The absence of transmembrane domains or GPI anchors in FGSE02 further supports its role as a secreted toxin, potentially targeting intracellular plant components to induce plant cell death. This hypothesis is reinforced by the yeast secretion assay confirming the functionality of its signal peptide, a signature of apoplastic effectors.

The time-course expression pattern of FGSE02 (peaking at 24 hpi) aligns with the essential transition from host penetration to colonization, a period where *F. graminearum* deploys virulence factors to evade plant immunity. The observed reduction in root infection severity in ΔFGSE02 mutants validates its significance during early infection. Intriguingly, FGSE02’s lack of impact on fungal growth contrasts with effectors like FGL1 [16], which are essential for both fungi virulence and development, suggesting functional specialization within the pathogen’s effector toolkit. This information implies that FGSE02 may act cooperatively with other virulence factors, such as DON toxin or cell wall-degrading enzymes, to optimize infection efficiency. For instance, DON’s role in suppressing host translation could amplify FGSE02-mediated cell death [47].

Despite these advances, several key issues remain scarcely understood. First of all, the molecular target of FGSE02 in plant cells is unknown. Future studies employing co-immunoprecipitation or yeast two-hybrid screens could identify interacting maize proteins, clarifying their mode of infection. Second, the conservation of FGSE02 homologs within Fusarium species raises questions about its evolutionary model and host specificity. Comparative genomic analyses across Fusarium strains infecting diverse crops may reveal adaptive variations in FGSE02 sequences linked to host range. Finally, ΔFGSE02 strains need to be evaluated in field trials under natural conditions to assess their practical significance in disease management strategies such as host-induced gene silencing or dsRNA gene silencing.

## 5. Conclusions

In summary, our work fills a critical gap in the understanding of *F. graminearum* effector biology by identifying FGSE02 as a cell death inducing virulence factor. While this study establishes its functional significance, further mechanistic and ecological investigations are essential to harness this knowledge for sustainable crop protection. These efforts could ultimately lead to novel control measures that reduce reliance on chemical fungicides and mitigate the global burden of Fusarium-related crop losses.

## Figures and Tables

**Table 1:** Basic information statistics of transcriptome sequencing data during *F. graminearum*–maize interactions.

**Sample**	**Clean Reads**	**Clean Bases**	**Read Length**	**Q20 (%)**	**GC (%)**	**Mapping to Fungi**
FG_fungal_1	82,073,226	12,310,983,900	150	96.91	51.85	97.91%
FG_fungal_2	81,677,390	12,251,608,500	150	97.15	51.90	98.05%
FG_fungal_3	81,157,202	12,173,580,300	150	97.13	51.84	97.56%
FG_24h_1	80,689,404	12,103,410,600	150	96.58	53.26	5.77%
FG_24h_2	81,429,004	12,214,350,600	150	97.12	53.66	5.82%
FG_24h_3	82,074,668	12,311,200,200	150	97.02	53.71	8.06%
FG_48h_1	80,281,800	12,042,270,000	150	96.49	53.62	22.06%
FG_48h_2	81,366,832	12,205,024,800	150	96.48	54.17	36.49%
FG_48h_3	79,535,168	11,930,275,200	150	96.41	53.44	17.25%
FG_72h_1	81,867,320	12,280,098,000	150	96.38	53.45	42.29%
FG_72h_2	77,635,306	11,645,295,900	150	96.42	53.80	37.64%
FG_72h_3	81,744,194	12,261,629,100	150	96.51	54.03	32.88%

## Data Availability

The original contributions presented in this study are included in the article/Supplementary Material. Further inquiries can be directed to the corresponding author.

## Supplementary Materials

The following supporting information can be downloaded at: https://www.mdpi.com/article/10.3390/jof11050397/s1. Figure S1: Evolutionary tree of FGSE02 and homologous proteins. Motif is a conserved sequence segment. Figure S2: Samples correlation heat map during *F. graminearum*-maize interactions. Figure S3: Relative expression level of *FGSE02*(*FG02*) at fungal, 24 hpi, 48 hpi, 72 hpi based on transcriptome data. Figure S4: Pathogenic phenotypes caused by *F. graminearum* wild type strain FG-12, ΔFGSE02 andΔFGSE02-C. Table S1: Up-regulated genes expressed in the early stage of *F. graminearum* infection (expression fold > 100). Table S2: Primers for Knockout Vector Construction. Table S3: primers for Plant expression vector construction. Table S4: primers for Yeast vector construction. Table S5: Gene expression per stage. Table S6: DON biosynthetic genes in FG12 genome. Table S7: Diseased region percent.
