# Supplementary material for: FGSE02, a Novel Secreted Protein in Fusarium graminearum FG-12, Leads to Cell Death in Plant Tissues and Modulates Fungal Virulence
Source: J Fungi (Basel). 2025 May 21;11(5):397. doi: 10.3390/jof11050397 (PMC12113282; doi:10.3390/jof11050397)
Supplement: Supplementary file 1 [file jof-11-00397-s001.zip › supplemental_Figure.pdf]

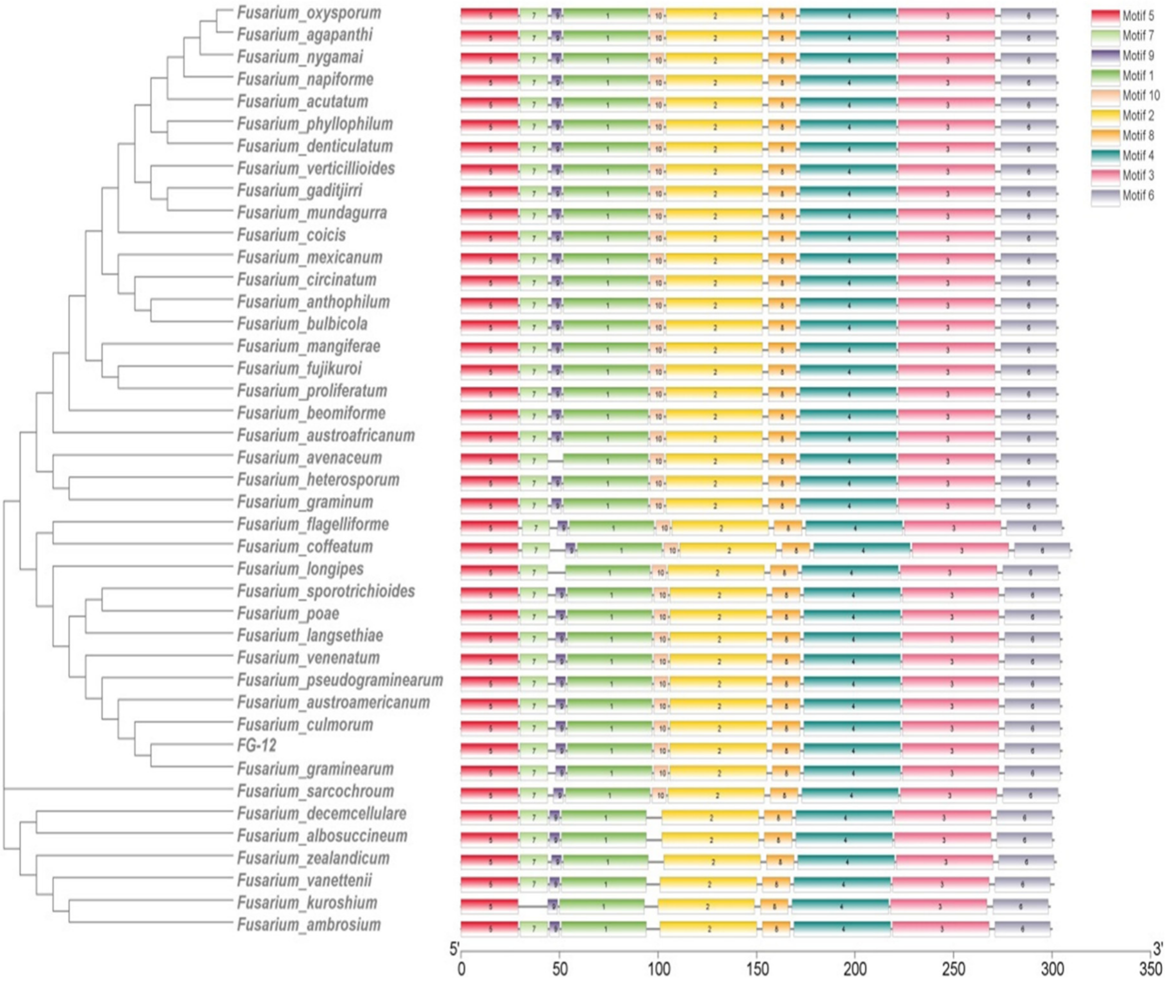

Figure S1. Evolutionary tree of FGSE02 and homologous proteins. Motif is a conserved sequence segment.

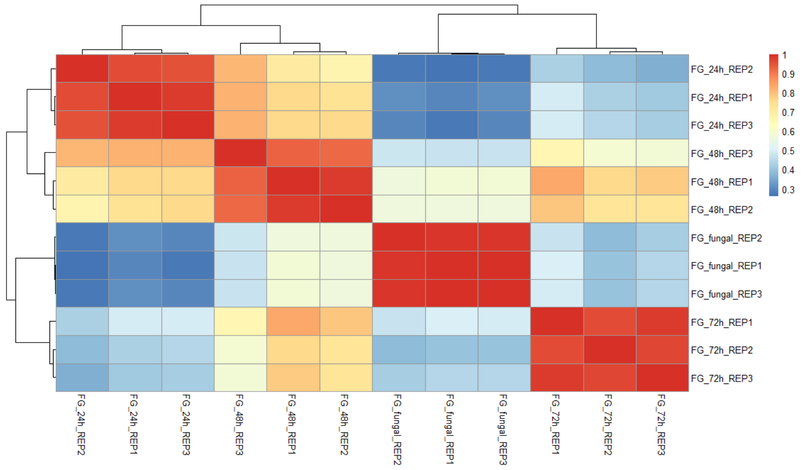

Figure S2. Samples correlation heat map during *F. graminearum*-maize interactions.

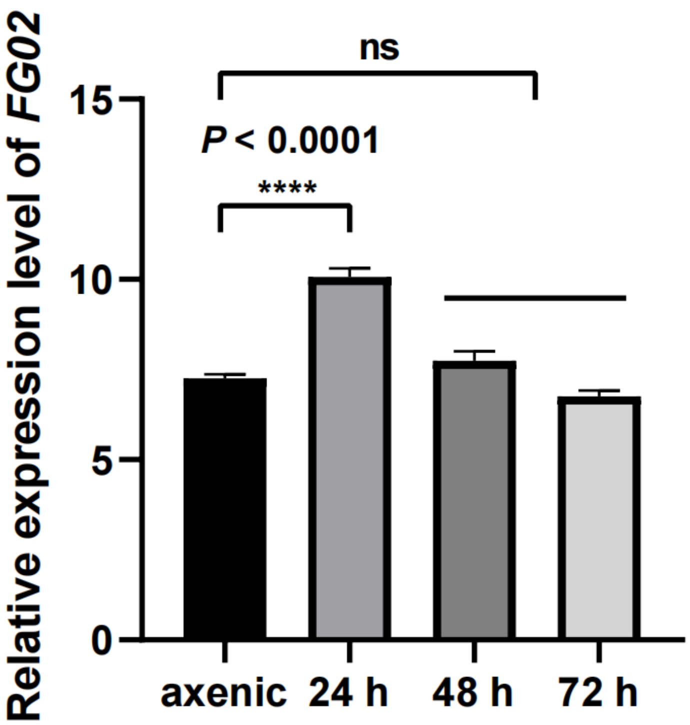

Figure S3. Relative expression level of *FGSE02*(*FG02*) at fungal, 24 hpi, 48 hpi, 72 hpi based on transcriptome data.

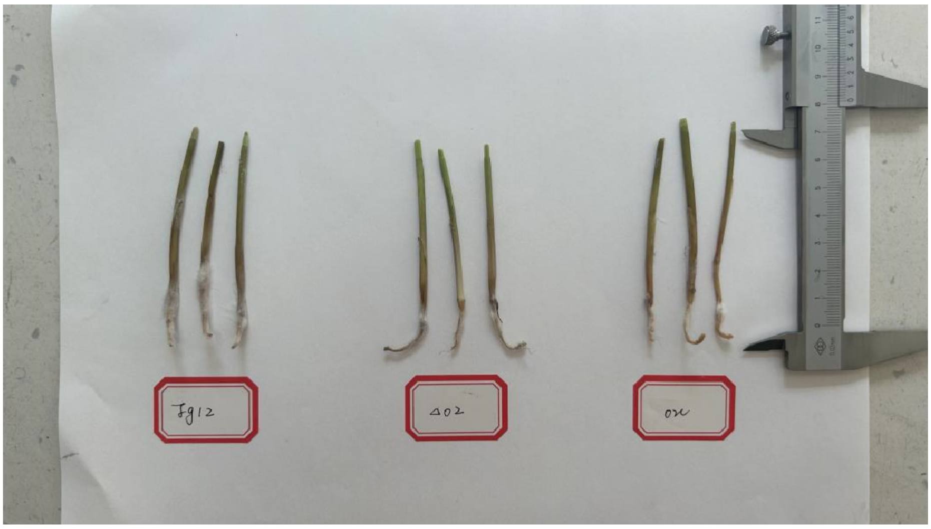

Figure S4. Pathogenic phenotypes caused by *F. graminearum* wild type strain FG-12, △FGSE02 and △FGSE02-C..
